# The Art of Bridge Building: A Look at the European-Level Cooperation in HTA (EU-HTA)

**DOI:** 10.3390/jmahp13020026

**Published:** 2025-05-28

**Authors:** Rui Santos Ivo, Tiago Rodrigues, Sara Couto, Mariane Cossito

**Affiliations:** 1 INFARMED—National Authority of Medicines and Health Products, I.P, 1749-004 Lisboa, Portugal; 2 Faculdade de Farmácia, Universidade de Lisboa, 1649-003 Lisboa, Portugal

## 1. Introduction

Health technology assessment (HTA) is a methodological and a scientific evidence-based process that allows competent authorities to determine the relative effectiveness of new or existing health technologies and to inform real-world decisions. HTA summarises information about medical, economic, social, and ethical issues related to the use of health technologies, such as medicinal products and medical devices [1].

The last few years have been marked by an increase in pharmaceutical expenditure that constrains the sustainability and affordability of health systems. This growth seems to be driven by many factors, such as novel and expensive therapies entering the market, the extension of indications leading to larger patient groups using costly therapies, demographic ageing, and the consequent increased impact of chronic diseases and subsequent use of medicines. Increases in prices—rather than an increase in volume—seems to be the main driver of pharmaceutical expenditure. HTA can be instrumental and contribute to the evidence base for selection and financing decisions related to medicines and health technologies, including price negotiations, thus significantly contributing to the greater sustainability of health systems [2,3].

Collaboration on HTA within Europe started in the mid-1990s and has evolved significantly in the decades since, moving towards the harmonisation of methodologies and procedures. The EUR-ASSESS project (1994), an initiative that laid the groundwork for future collaborations, HTA-Europe (1997), ECHTA/ECAHI (2000–2002), and the European Network for Health Technology Assessment (EUnetHTA) joint actions and projects (2006–2023). All these projects and joint work provided comprehensive recommendations for HTA in relation to policy and practice, the sharing and development of methodological and practices, joint assessments, information exchange, and education and training, thus forming the foundations for cross-border collaboration in HTA in Europe [4,5].

The establishment of EUnetHTA, which promoted joint clinical assessments and joint scientific consultations, thereby facilitating knowledge sharing and establishing best practices among over 70 agencies, was of the utmost importance and can be perceived as the precursor of the HTA system established by the EU HTA Regulation (Regulation (EU) 2021/2282 on HTA (HTAR)) adopted in 2021 [1].

On the other hand, it is important to recall that the Cross-Border Directive (Directive 2011/24/EU on cross-border healthcare) provided the political and regulatory framework for joint actions under the EUnetHTA, stating in article 15 that “*The Union shall support and facilitate cooperation and the exchange of scientific information among Member States within a voluntary network connecting national authorities or bodies responsible for health technology assessment designated by the Member States*”. The Directive provided a detailed legal framework focused on rules concerning the reimbursement of cross-border healthcare cost, cross-border healthcare responsibilities of Member States and cooperation between healthcare systems. Although it was not, in essence, its purpose, the Directive laid a legal framework that supported cooperation between national HTA authorities, which was pivotal for the launch of the aforementioned initiatives, but also encouraged the creation and promotion of others such as the Baltic Procurement Initiative, BENELUXA Initiative, Fair and Affordable Pricing, Nordic Pharmaceutical Forum, and La Valletta Declaration. This further strengthened collaboration on price and reimbursement and accessibility.

## 2. EU HTA Regulation: The Cornerstone of the European Health Technology Assessment Collaboration

The adoption of the EU HTA Regulation is a major milestone of the utmost importance that established a legal and financial framework for European-level cooperation in HTA (EU-HTA), with a phased implementation starting from January 2025. The system sets in place common methodologies and procedures for clinical assessments and scientific consultations and also creates the conditions for voluntary cooperation in HTA and in non-clinical areas of HTA. This regulation aims to improve the quality and efficiency of health interventions and ensure the sustainability of health systems across Europe [1]. In the long term, the EU HTA Regulation is expected to eliminate the duplication of efforts on the transferable aspects of HTA reports, such as evidence on relative effectiveness and relative clinical safety and deliver efficiency gains in the lead-up to national decisions on pricing and reimbursement and facilitate faster and more equitable access to medicines and other health technologies.

There are fundamental principles underlying the functioning and future success of the European HTA collaboration: solidarity between Member States in working together and sharing knowledge and responsibilities, trust and reliability in each other’s work and the art of building bridges, between agencies and with stakeholders. These were also the principles that guided the construction of the EU regulatory system for medicines. Once again, this aspect will be crucial to foster connections amongst those involved. We can learn from the experience of regulatory authorities and the way the European medicines regulatory network works and cooperates. Furthermore, it is important to note that among the health technology assessment bodies, there are some with competences and attributions in the regulatory area, which can provide a competitive edge and be advantageous towards achieving successful outcomes of joint work expected by 2030 [6].

## 3. Key Challenges and Opportunities in Building and Consolidating the European Health Technology Assessment System

One of the primary challenges faced by HTA in Europe is the heterogeneity of conditions and needs—within different national health systems—leading to the fragmentation of processes and methodologies across different countries. This diversity can lead to differences in assessment outcomes, hindering harmonised decision making and the uniform implementation of new health technologies. Additionally, the increasing complexity of technologies, ranging from biological treatments to digital interventions, requires an interdisciplinary approach as well as robust adaptable evaluation methodologies. The implementation of the EU HTA Regulation therefore presents several opportunities. Based on previous harmonisation and experience, particularly the EUnetHTA’s joint actions and projects, the Member State Coordination Group on HTA (HTACG) has been working on a common evaluation framework, ensuring that all health technologies are assessed consistently and based on solid evidence. More than a dozen scientific, methodological, and practical guidance documents for joint clinical assessments and joint scientific consultations have been developed and made public by the HTACG in a herculean effort to set in place a common and harmonised framework. It is important to highlight that these efforts were Member State-driven.

For the success of the regulation and the activity it establishes, the HTA IT platform (currently in development by the European Commission) will be pivotal in guaranteeing the efficiency, quality, and accuracy of assessments.

## 4. Improving Interactions Between HTA Bodies and Regulatory Authorities

The Council of Health Ministers on access to medicines and medical devices for a stronger and more resilient EU (June 2021) identifies evidence generation as an area for the future, optimising data collection, taking advantage of the digital transformation, and cooperating to transform data into evidence that supports regulators, health technology assessment, payers, clinical decision-makers, and patients towards improving efficiency and health outcomes.

Along the same line, the need to optimise drug development and facilitate faster access for patients has led to discussions about the importance of building bridges and improving interactions between HTA bodies and regulatory authorities. There are several areas where an alignment of evidentiary requirements on both perspectives can occur, e.g., the choice end point and use of surrogate measures, the inclusion of active comparator arm in trial, and the definition of unmet medical need [7].

## 5. Cooperation and Coordination

The COVID-19 pandemic was one of the longest and most intense health crises the EU and its Member States have ever had to face. This collective experience and the current and possible health global challenges have highlighted the importance of cross-country cooperation and collaboration between regulators, health technology assessment, payers, clinical decision-makers, and patients.

The EU HTA Regulation sets in place a clear governance with balanced Member State-driven participation, characterised for being Member-State drive.Therefore, the HTACG will play a central and fundamental role in planning and coordinating the foreseen joint work.

The Heads of HTA Agencies Group (HAG) has also contributed to the good governance of the European HTA collaboration. The HAG is an independent forum for raising, highlighting, and discussing strategic topics and matters are relevant to HTA bodies across Europe. Established in 2021, the HAG comprises more than 30 European healthcare agencies from more than twenty EEA/EU Member States working together to advance strategic collaborations on HTA. The HAG has been supporting the development basis for the joint work that will be undertaken on all HTA activities at the EU level within the model of EU cooperation, as anticipated by the EU HTA Regulation, and the preparation of national systems and capacity for the adoption and joint work conducted under the HTA Regulation [8].

Finally, it is worth emphasising the EU HTA Regulation foresees voluntary cooperation in non-clinical areas of HTA and in technologies that are out of scope of the EU HTA, which can contribute to the harmonisation and sharing of knowledge regarding HTA.

The Joint Nordic HTA Bodies (JNHB) and BeNeLuxA are two of the many initiatives worth following over the next few years, along with other regional collaborations that will certainly form in turn over the next few years. In addition, there are other cross-border collaborations that will contribute to a more cohesive Europe and greater cooperation between the different agents and players in the sector, ultimately contributing to a faster access to therapeutics with benefit or clinical added value, such as the International Horizon Scanning Initiative (IHSI) and National Competent Authorities on Pricing and Reimbursement (NCAPR) group.

## 6. Resource Allocation and Capacity Building

The development of HTA and related activities (e.g., managed entry agreements and horizon scanning processes) requires specialised knowledge and skills for the evaluation of emerging and complex technologies and capable resources, all of which demand substantial and sustained financial investment [9,10]. The need to strengthen HTA bodies’ operational and resource capacity—and of course the national legal and regulatory framework that determines the activity and work of an HTA body in each country, as well as their adaptation to the new EU HTA Regulation—have been identified as priorities for the coming months.

A European consortium financed by EU funds—the Head of Agencies Group Initiative for Knowledge and Skill Enhancement in Health Technology Assessment Regulation (HAG-INSIGHT)—was announced at the end of 2024. The initiative aims “*to strengthen the long-term capacity and expertise of EU HTA bodies in executing the HTAR effectively and in a sustainable manner in view of the upcoming health technology scope expansions*”. Moreover, the project will “*establish a comprehensive HTAR training platform on procedures and methods with latest insights*” [11]. In addition to these, several other initiatives targeted at capacity building are now emerging.

## 7. Conclusions

The EU HTAR is a major milestone on public health and HTA collaboration in Europe and is expected to change the HTA landscapeIt can become a world reference for HTA activities and collaboration [12].

By addressing the current challenges and seizing the available opportunities, Europe can establish a long-term HTA collaboration that can contribute by improving efficiency in the uptake of pharmaceuticals by health systems following their marketing, improving health outcomes, promoting sustainability, and ultimately promoting European competitiveness. Collaboration, harmonisation, and strategic investments are thus essential to achieving this goal.

In the coming months, we will see the progressive implementation of the EU HTA Regulation. Special attention will be paid to the challenges encountered during the process, but also to monitoring the implementation and application of the results from joint clinical assessments and joint scientific consultations and other joint work conducted under the regulation, and assessing financing and pricing decisions and access to medicinal products and medical devices at the national level [13].

Long-term EU HTA collaboration will require that we all deepen our skills in the art of bridge building.
